# Linking Immunoevasion and Metabolic Reprogramming in B-Cell–Derived Lymphomas

**DOI:** 10.3389/fonc.2020.594782

**Published:** 2020-11-05

**Authors:** Martin Böttcher, Rebecca Baur, Andrej Stoll, Andreas Mackensen, Dimitrios Mougiakakos

**Affiliations:** Department of Medicine 5 for Hematology and Oncology, Friedrich-Alexander-Universität Erlangen-Nuremberg, Erlangen, Germany

**Keywords:** B-cell-derived Non-Hodgkin lymphoma, chronic lymphocytic leukemia, metabolism, immune escape, immune therapeutics

## Abstract

Lymphomas represent a diverse group of malignancies that emerge from lymphocytes. Despite improvements in diagnosis and treatment of lymphomas of B-cell origin, relapsed and refractory disease represents an unmet clinical need. Therefore, it is of utmost importance to better understand the lymphomas’ intrinsic features as well as the interactions with their cellular microenvironment for developing novel therapeutic strategies. In fact, the role of immune-based approaches is steadily increasing and involves amongst others the use of monoclonal antibodies against tumor antigens, inhibitors of immunological checkpoints, and even genetically modified T-cells. Metabolic reprogramming and immune escape both represent well established cancer hallmarks. Tumor metabolism as introduced by Otto Warburg in the early 20th century promotes survival, proliferation, and therapeutic resistance. Simultaneously, malignant cells employ a plethora of mechanisms to evade immune surveillance. Increasing evidence suggests that metabolic reprogramming does not only confer cell intrinsic growth and survival advantages to tumor cells but also impacts local as well as systemic anti-tumor immunity. Tumor and immune cells compete over nutrients such as carbohydrates or amino acids that are critical for the immune cell function. Moreover, skewed metabolic pathways in malignant cells can result in abundant production and release of bioactive metabolites such as lactic acid, kynurenine or reactive oxygen species (ROS) that affect immune cell fitness and function. This “metabolic re-modeling” of the tumor microenvironment shifts anti-tumor immune reactivity toward tolerance. Here, we will review molecular events leading to metabolic alterations in B-cell lymphomas and their impact on anti-tumor immunity.

## Introduction

Metabolic reprogramming is a well-established hallmark of cancer (1). In fact, emerging evidence suggests that metabolic reprogramming does not only confer bioenergetic advantages but also impacts immune surveillance, thus being closely interconnected with immune escape, another hallmark of cancer (1). In this mini-review on B-cell-derived lymphomas, we will focus on metabolic alterations, the underlying molecular mechanisms, and their impact on anti-tumor immunity.

In general, cells meet their energetic demands in form of adenosine triphosphate (ATP) by different degrees of either glycolysis or mitochondrial oxidative phosphorylation (OxPhos). Although both metabolic axes happen simultaneously, there is a distinct fine-tuning of the balance between glycolysis and OxPhos. Non-proliferating, quiescent or differentiated cells primarily rely on OxPhos in the presence of oxygen and only switch to glycolysis under hypoxia. Highly proliferative cells obtain most of their ATP by aerobic glycolysis despite the availability of oxygen, a mechanism introduced by Otto Warburg in the 1920s and referred to as the “Warburg effect” (2).

Although aerobic glycolysis is less efficient, energy generation is faster and provides intermediates such as nucleotides, amino acids, and carbons for fatty acid (FA)/lipid synthesis; all crucial components for dividing cells. Consequently, aerobic glycolysis is predominantly found in activated immune cells such as effector T-cells (3) but also in most dividing malignant cells (4).

OxPhos provides an enhanced metabolic flexibility as it can be fueled by different sources, primarily glucose, glutamine, and FAs. Cell types that preferentially utilize OxPhos are slow-dividing, long-lived immune cells such as regulatory and memory T-cells (5) or leukemia stem cells (6).

Apart from the energy provision, metabolic processes impact a plethora of cellular functions by amongst others interfering with translation, epigenetics, and post-translational modifications as reviewed by Patel et al. (7). As such, metabolism represents a central regulator of cell fate and function.

Owed to its vital importance metabolism is tightly regulated by both intrinsic and extrinsic mechanisms. Malignant cells display aberrations in those regulatory circuits leading to metabolic profiles that favor survival, growth, and immune escape. In the follow, we will discuss the most prominent metabolic alterations described in B-cell-derived Non-Hodgkin lymphomas (B-NHLs) including chronic lymphocytic leukemia (CLL).

## Metabolic Alterations in B-NHLs and CLL

### Diffuse Large B-cell Lymphoma (DLBCL)

As the most common form of B-cell lymphomas, aggressive DLBCL accounts for about 35% of all newly diagnosed B-NHLs (8). The DLBCL displays a pronounced heterogeneity in terms of genetic background and outcome. The cell of origin (COO) algorithm categorizes DLBCLs based on the gene expression profiles (GEPs) into “germinal center B-cell like” (GCB) types resembling normal germinal center B-cells and “activated B-cell like” (ABC) types with GEPs reminiscent of *in vitro* activated B-cells (9–11). An additional classification framework known as consensus cluster classification (CCC) revealed three separate clusters with distinct metabolic fingerprints: OxPhos-DLBCL, B-cell receptor (BCR)-DLBCL, and host response (HR)-DLBCL (12). OxPhos-DLBCLs display a prominent mitochondrial component, with elevated OxPhos, an overall increased mitochondrial contribution to the total energy turnover, and a greater incorporation of carbons derived from FAs and glucose into the tricarboxylic acid (TCA) cycle. In contrast, non-OxPhos-DLBCLs are metabolically rewired toward aerobic glycolysis (13). Immunohistochemical studies in DLBCL revealed expression of transporters of lactate (i.e., MCT1 and TOMM20) that can fuel the TCA cycle of malignant cells in a process better known as the “reverse Warburg effect” (14). Interestingly, OxPhos-DLBCL exhibited marked susceptibility toward inhibition of mitochondrial FA oxidation (FAO) and of PPARγ that regulates FA uptake and storage (13). BCR-DLBCLs were susceptible to pharmacological SYK inhibition (15), which in turn leads to a downregulation of glycolytic components (such as GLUT1 and hexokinase 2) (16).

As a central hub for the integration of metabolic processes, mammalian target of rapamycin (mTOR) controls nutrient/amino acid sensing, glycolysis, OxPhos, and consequently proliferation and survival. It serves as the core component of two multi-protein complexes (mTORC1 and mTORC2) that regulate different cell processes [reviewed in (17)]. Non-GCB DLBCLs depict increased mTOR-activity, which is linked to inferior survival (18). However, an *in vitro* study conducted on different DLBCL cell lines demonstrated therapeutic efficacy of mTOR inhibitors independent of COO. Overall, no clear link between COO- or CCC-based classifications and mTOR activity could be established yet.

Furthermore, DLBCLs (over-)express indoleamine-2,3-dioxygenase (IDO), which catalyzes breakdown of the essential amino acid L-tryptophan into the catabolite L-kynurenine (19). The latter one could promote expression of the pro-oncogenic Bcl-6 in DLCBL (20). In fact, both enhanced IDO expression (21) as well as elevated serum L-kynurenine levels (22) were linked to reduced response rates and inferior 3-yr overall survival (OS).

### Follicular Lymphoma (FL)

The second most common type of B-NHL is the indolent follicular lymphoma (FL) (23). In FL SYK is, similar to DLBCL, highly activated and regulates mTOR (24). In addition, recurrent somatic mutations of *RRAGC* that encodes for the Ras-related GTP-binding protein C are the leading cause for mTORC1 activation in FL (25) and render FL cells more susceptible toward mTOR-induced cytotoxicity (26). Beyond that, metabolism of FLs remains largely unexplored. Notably, transformation into DLBCL is associated with an enhanced expression of the glycolytic machinery, which is in line with the increased glucose uptake as revealed by ^18^F-FDG PET/CT in transformed lymphomas (27, 28).

### Mantle Cell Lymphoma (MCL)

MCL represents about 5-10% of B-NHLs. Despite being classified as indolent, it has a rather aggressive disease course. MCL cell lines display constitutive mTOR activation (29). A dysregulation of the upstream PI3K/AKT pathway has been implicated as a driver of mTOR in MCL. This notion is further corroborated by the observation that the phosphatase and tensin homologue (PTEN), which acts as an intrinsic PI3K/AKT inhibitor, can be reduced or undetectable in MCL (30). Inhibiting mTOR was effective in targeting MCL metabolism (31) and is approved for the relapsed/refractory (r/r) situation based on positive data from clinical studies (32). The Bruton tyrosine kinase (BTK) inhibitor ibrutinib abolishes BCR signaling and has emerged as a potent therapeutic option for r/r MCL. BTK-blockade markedly affected the (ibrutinib-responsive) MCLs’ metabolic activity including glycolysis and the TCA cycle (33). Interestingly, Zhang et al. reported that ibrutinib-resistant MCLs depict a metatobolic rewiring toward glutaminolysis-fueled OxPhos (34). These drug-resistant cells were readily targeted by a OxPhos inhibitor, showing promising efficacy in patient-derived preclinical models.

### Chronic Lymphocytic Leukemia (CLL)

CLL as the most common adult leukemia of the Western world is a heterogeneous disease characterized by accumulating monoclonal B-lymphocytes (35). Circulating CLL cells are mainly quiescent and proliferation predominantly occurs in lymph nodes (LNs) and the bone marrow (BM). Nevertheless, circulating CLL cells possess a marked metabolic activity that differs from healthy B-lymphocytes. As they traffic between hypoxic (i.e., LN and BM) and normoxic compartments (i.e., peripheral blood), CLL cells were found to constitutively express hypoxia-inducible factor (HIF-1α), which gets further upregulated within LNs thus promoting aerobic glycolysis (36, 37). Hypoxia-induced upregulation of glycolytic genes is further supported by adenosine signaling, which is triggered by the CLL cells’ ectonucleotidases CD39 and CD73 (37).

CLL cells *per se* contain more mitochondria than conventional B-lymphocytes. Endogenous nitric oxide (NO) levels correlate positively with mitochondrial mass (38). In fact, NO can drive mitochondrial biogenesis, as NO supplementation increases mitochondrial mass in B-NHL-derived cell lines, whereas NO inhibition antagonizes this process (39). Correspondingly, Jitschin et al. demonstrated enhanced mitochondrial OxPhos, respiration, and respiratory capacity (40). The thereby amplified electron turnover *via* the mitochondrial electron transport chain yielded increased levels of ROS within the CLL cells but also systemically. Oxidative stress led then to a compensatory upregulation of heme-oxygenase-1 in CLL cells, a key cellular antioxidant, which also functions as a positive switch for the key activator of mitochondrial transcription factor A. Mitochondrial biogenesis, increased respiration, and oxidative stress appear to form a positive self-reinforcing feedback loop. As previously shown for solid tumors, pyruvate can act as a scavenger of mitochondrial superoxide in CLL cells. Increased oxidative stress under hypoxia led to enhanced pyruvate uptake while normoxic conditions led to a pyruvate release (36).

In fact, CLL cells from patients with higher disease stages and those with molecular features associated with a poor prognosis, like unmutated IGHV (U-CLL) and ZAP-70 positivity, showed higher mitochondrial respiration (41, 42). Both aforementioned genetic risk factors foster BCR signaling. Consistently, BCR-targeting reduces the metabolic activity (41). In general, CLL cells and conventional B-cells did not differ in terms of their basal glycolytic rate. However, CLL cells showed an elevated glycolytic capacity and glycolytic (together with respiratory) reserve. Furthermore, patients with U-CLL had higher lactic acid serum concentrations and displayed an elevated glycolytic capacity as compared to their mutated counterparts (M-CLL) (43). This suggests that CLL cells are better equipped to adapt to fluctuations of bioenergetic resources. In fact, microenvironmental stimuli further support the CLL cells’ metabolic flexibility as contact to LN- or BM-resident stromal cells elicits a glycolytic switch in a Notch/Myc-dependent manner (44). Transferring these findings into the clinics, patients with higher glycolytic capacity and reserve have a worse OS and a shorter time-to-treatment (43). Furthermore, CLL samples with higher glycolytic flexibility showed an increased resistance to novel drugs affecting the mitochondria, such as venetoclax and navitoclax (43).

Another metabolically important aspect is the role of free fatty acids (FFAs). Lipoprotein lipase (LPL) is the major enzyme catalyzing hydrolysis of triglycerides into FFAs and is mainly present in adipose tissue, playing a key role in lipid metabolism. CLL cells carry LPL on their cell membrane, while LPL gene expression is elevated in U-CLL cells (45, 46). LPL facilitates lipoprotein uptake, which enables CLL cells (unlike normal B-cells) to store and metabolize FFAs (46). Intracellular FFAs can then be used to promote the already more active OxPhos in CLL cells (47). Moreover, FFAs may themselves drive mitochondrial biogenesis through activation of PPARγ (48). Upregulation of LPL in CLL cells is at least partly mediated by STAT3, since STAT3 binds to the LPL promotor and STAT3 knockout downregulated LPL protein levels (46). In addition to LPL, CD36 (a cell surface FA translocase) density is higher on CLL cells as compared to non-malignant B-cells. CD36 expression (and the consecutive FA uptake) were driven by STAT3 and inhibition of FA uptake reduced CLL cell viability (49). Again, BTK-inhibition reduced LPL levels and FFA metabolism both *in vitro* and in patients at least partly through interfering with STAT3 (50). Interestingly, CLL ibrutinib-resistant cases could be targeted by an FAO-inhibitor highlighting the importance of FAO metabolism and OxPhos (51). Emphasizing the relevance of FFA metabolism in CLL, the OS of patients with high LPL levels was worse than for those with low ones (45).

**Figure 1:**
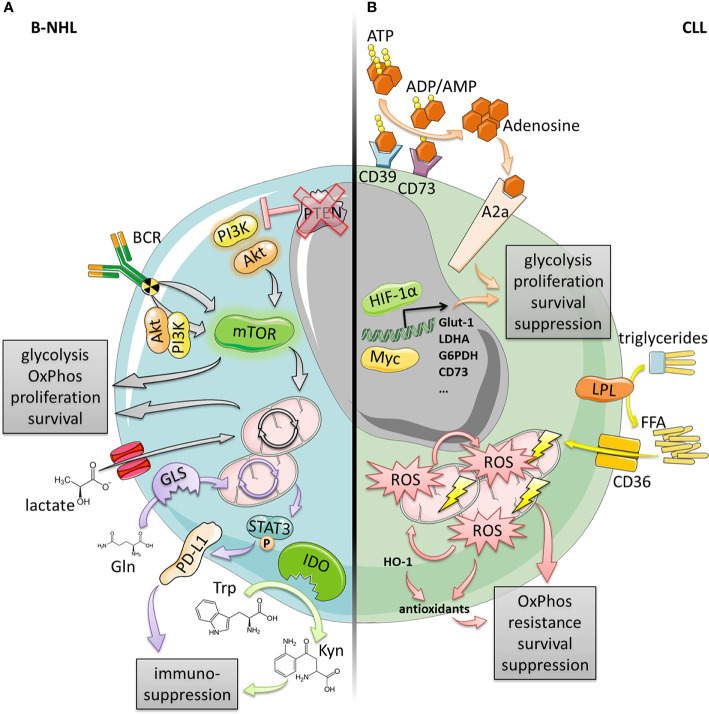
Metabolic alterations in B-cell lymphomas and CLL. **(A)** B-NHL (DLBCL, MCL, FL) often exhibit an elevated mTOR signaling activity enabling increased glycolysis, OxPhos, proliferation and survival. This can be driven by the BCR in a PI3K/AKT-dependent or independent manner, or by genetic events such as the loss of PTEN expression resulting in constitutively active PI3K/AKT. Increased OxPhos was also found to be fueled by elevated lactate shuttling into the TCA cycle due to increased lactate importer expression. Additionally, both increased expression of IDO as well as glutaminolysis-driven PD‑L1 induction provide enhanced immune-suppression. **(B)** CLL cells display a high mitochondrial biomass and high levels of OxPhos generating large amounts of energy and ROS that in turn drive mitochondrial biogenesis and generation of antioxidants (at least partly by HO-1). This vicious cycle confers enhanced oxidative stress resistance, survival and suppression. OxPhos and mitochondrial biogenesis could also be driven by increased activity of LPL and CD36 consuming triglycerides and importing free fatty acids (FFA). In contrast, microenvironmental trigger (e.g., hypoxia or LN-/BM-stroma) can induce transcription factors, such as Myc or HIF-1α leading to a glycolytic switch (enabled by high metabolic flexibility), and an increase of the adenosinergic axis culminating in enhanced survival, proliferation, and suppression.

Taken together, metabolic adaptations and flexibility occur in multiple facets in DLBCL, FL, MCL, and CLL (**Figure 1**). They confer enhanced survival, proliferation, and therapeutic resistance but at the same time, we can therapeutically exploit them.

## Lymphoma Metabolism and Its Potential Impact on Anti-Lymphoma Immunity

**Figure 2:**
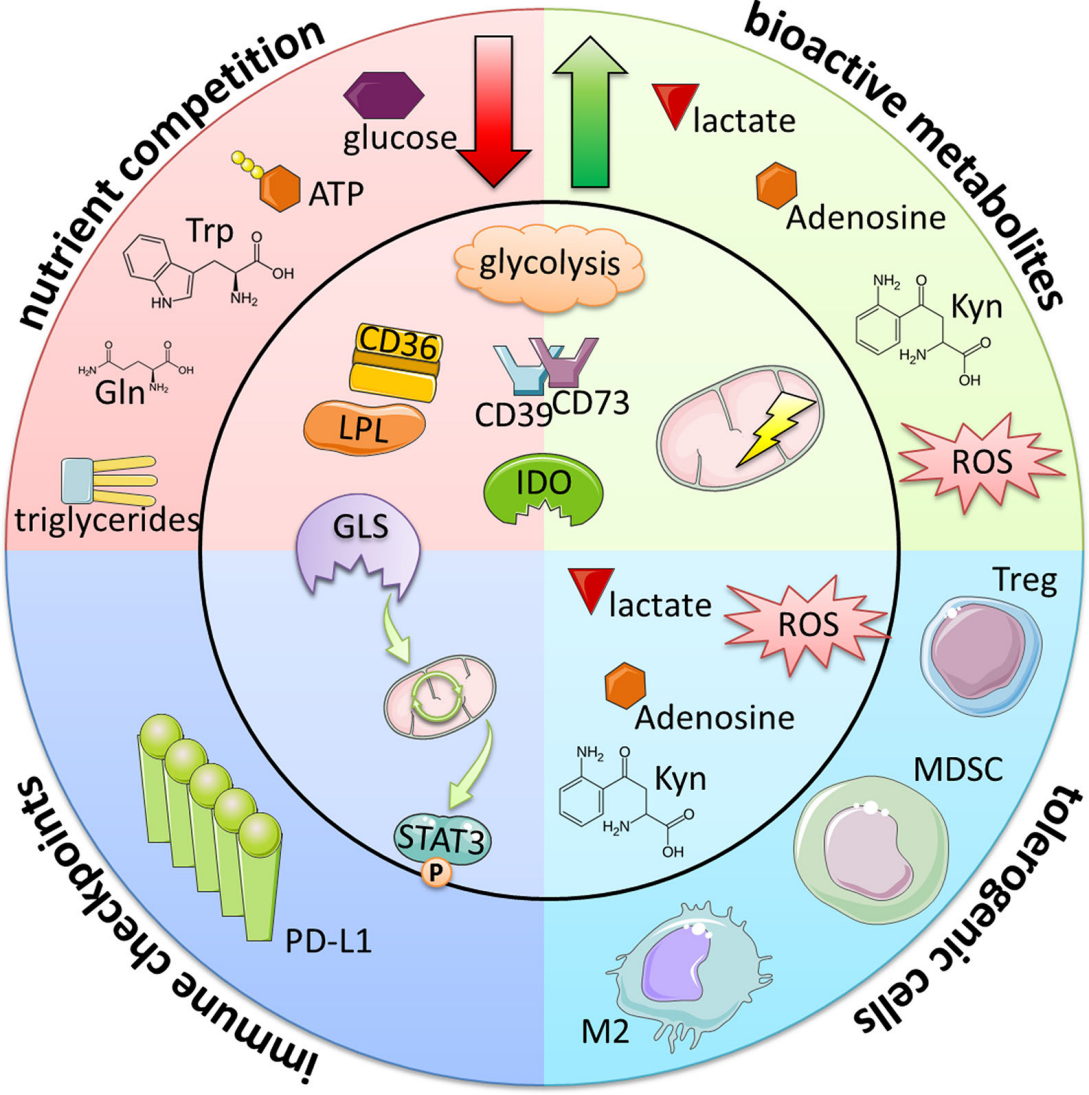
Immunometabolic evasion mechanisms of B-cell lymphomas and CLL. Metabolic alterations lead to the reduction of essential immune cell nutrients and at the same time to the generation of bioactive metabolites. In addition to their direct immuno-suppressive nature, these byproducts also increase the frequencies of tolerogenic, suppressive immune cells (such as Tregs, myeloid-derived suppressor cells [MDSCs] and M2-like macrophages) either indirectly *via* the creation of favorable conditions or directly inducing their differentiation. Furthermore, certain metabolic programs can promote the expression of immune checkpoints such as PD-L1. Taken together, these events ultimately lead to the overall suppression of immune attack and increase the tumor cells’ survival and persistence.

Tumors including B-cell malignancies have developed a variety of mechanisms to evade the anti-tumor immunity. Amongst others, four major “metabolic strategies” have been identified (**Figure 2**): 1) competition over nutrients, 2) production of bioactive metabolites, 3) induction/promotion of regulatory, tolerogenic immune cells, and 4) metabolic control of immune checkpoints (ICPs).

The most studied example of nutrient competition is the increased glucose consumption by malignant cells caused by elevated expression levels of glucose transporters and enzymes of the glycolytic machinery {as seen in BCR-DLBCL [e.g., GAPDH expression (52) and lactate secretion (13)], transformed FL [e.g., GAPDH and aldolase A (27, 28)], MCL [e.g., glycolytic flux (33)], and CLL in the LN-/BM-niche [e.g., glycolytic flux and key glycolytic enzymes (44)]}. This is detrimental for T- and NK-cells as their proliferation, activation, and differentiation is highly dependent on glucose as a fuel for both aerobic glycolysis and OxPhos [reviewed in (53)]. Similarly, tumoricidal (M1) macrophages depend on glucose to fully mount their effector cytokine response [reviewed in (54)]. Additionally, increased activity of lipid/FFA-consumptive enzymes (i.e., LPL and CD36 as seen in CLL) as well as of glutaminase (as seen in DLBCL) contribute to a nutrient-poor environment. Apart from the depletion of basic bioenergetic substrates, increased expression of the ectonucleotidases CD39/CD73 (as seen in CLL) reduces extracellular ATP (exATP) by enzymatic conversion to adenosine. exATP was shown to be particularly important for long-lived T-cell immunity through purinergic signaling promoting mitochondrial fitness (55). Expression of L-tryptophan-depleting IDO (as seen in DLBCL) has been linked to inferior progression free survival and OS in B-cell malignancies (21). In T-cells L-tryptophan shortage leads to cell cycle arrest (56) at least partly due to a stress response caused by uncharged transfer-RNAs (57), and to reduced proliferation caused by mTOR inhibition (58). Furthermore, tryptophan is essential for clonal expansion and effector T-cell differentiation *via* metabolic reprogramming through mTOR (59).

Simultaneously, the different rewired metabolic activities lead to accumulation of various bioactive (and potentially immunomodulatory) metabolites within the tumor microenvironment (TME). This includes lactate (as a result of enhanced glycolytic activity) which leads to the inhibition of T- and NK-cells *via* blunted lactate export acidifying the cytoplasm (60) as well as reduced NFAT levels (61) resulting in diminished cytokine production and effector function. Similarly, the IDO catabolite L-kynurenine inhibits immune-cell function (62), e.g., by induction of T-cell exhaustion (63, 64) and deregulation of NK-cell activating receptors (65). In fact, increased L-kynurenine serum concentration is associated with inferior OS in DLBCL (22). Accumulating extracellular adenosine converted from exATP by CD39/CD73 (as seen in CLL) can blunt immune responses by activating adenosine receptors that signal *via* cyclic AMP and protein kinase A. T-cells respond with reduced proliferation (66, 67), NFκB activity (68), and cytokine production (67, 69, 70) as well as increased exhaustion (71). NK cells are similarly affected by adenosine (67, 72). Abundant ROS (as seen in CLL, DLBCL, and FL) regularly leads to oxidative stress in malignancies. Again, T- and NK-cells are particularly sensitive toward ROS-induced cytotoxicity (73, 74), e.g., through impairment of T-cell receptor signaling (75, 76) leading to reduced cytokine production (77). This is reflected by the negative prognostic impact of oxidative stress in DLBCL (78) and FL (79).

However, those bioactive metabolites are not only capable of direct immune cell suppression, but can also favor preferential survival and/or induction of tolerogenic cell types. Actually, regulatory T-cells (Tregs), myeloid-derived suppressor cells (MDSCs), and pro-tumorigenic M2-like macrophages accumulate in B-cell-derived malignancies. As a prime example, kynurenine directly promotes reprogramming toward Tregs by inducing their master transcription factor FOXP3 (80). At the same time, Tregs are more resistant than conventional (potentially tumor-directed) T-cells toward detrimental effects caused by abundant lactate (81) or ROS (82) thereby enjoying a survival benefit.

As stated above, a novel, fourth mechanistic axis has been established interconnecting metabolic activity and expression of ICPs such as PD-L1 that prevent mounting of an effective anti-tumor immunity. PD-L1 is found in B-NHL and can have a negative prognostic impact (e.g., for DLBCL) (83, 84). Consequently, ICP blockade is currently undergoing clinical evaluation. Recent studies have demonstrated that glucose uptake and glutaminolysis are required for a stable PD-L1 expression. In DLBCL, glutaminolysis contributes to STAT3 induction, which positively regulates PD-L1 (85). Glucose serves as a substrate for posttranslational protein glycosylation, while N-glycosylation of PD-L1 maintains its stability and interaction with its cognate receptor (86).

Overall, metabolic reprogramming is closely linked to immunoevasion. However, many of the here described phenomena are extrapolated from basic studies or translational research within different disease contexts. Thus, immunometabolic research in B-cell malignancies needs to be further extended to build a sound basis for novel treatment strategies.

## Conclusion and Future Perspectives

The concept of immunometabolic regulation has emerged as an important research field. Interplay between cells does not only occur *via* signaling molecules and receptor-ligand interactions but also through metabolic communication. Tumor cells have adapted their metabolic regulatory circuits (**Figure 1**), which improves their survivability and resistance toward anti-tumor immunity and/or (immune-based) therapies (**Figure 2**). However, metabolic reprogramming can cause novel metabolic dependencies and/or vulnerabilities rendering malignant cells more susceptible toward interferences within their metabolic framework as already described for DLBCL (13, 15), FL (24, 26), MCL (31, 32) and CLL (41, 43, 50, 51).

Targeting key (dysregulated) metabolic molecules would represent one very obvious strategy for re-establishing a (for immune cells) more favorable environment without lack of nutrients or presence of detrimental catabolites. However, it needs to be taken into consideration that bioenergetic processes of malignant cells and of activated immune cells are very similar. As such, mTOR as a central hub for nutrient sensing and bioenergetic regulation in various types of B-NHLs would represent a *bona fide* target. Activated T-cells are also strongly dependent on mTOR-regulated uptake of glucose and amino acids [reviewed in (87)]. Thus, targeting mTOR would inevitably affect the T-cell’s metabolic competence (and consequently anti-tumor function) as seen in preclinical models (88). Here, focusing on pathways that are not directly associated with the cell’s bioenergetics and self-evidently do not overlap between malignant and immune cells is more promising. Blockade of IDO (that is not expressed in T- and/or NK-cells) for example is currently investigated in a number of malignancies (89). Notably, reducing ROS production (by histamine application) coupled with IL-2-triggered T- and NK-cell activation has led to solid clinical effects in patients with acute myeloid leukemia (90) and comparable observations were reported when combining bicarbonate that neutralizes an acidic milieu with ICP blockade in preclinical melanoma models (91).

Nowadays, adoptive transfer of genetically engineered chimeric antigen receptor (CAR) T-cells has heralded a new era in the immunotherapy of cancer in particular of B-NHLs (92). The efficacy of CAR T-cell treatment and their adequate anti-tumor effect rely on sustained metabolic activity and energy supply as well as *in vivo* persistence. In analogy to the intrinsic anti-tumor immune responses, the TME can represent a metabolic barrier for CAR T-cells as convincingly shown for IDO and anti-CD19 CAR T-cells in a preclinical lymphoma model (93). Manipulating the metabolic equipment of CAR T-cells itself to empower their function in the TME [reviewed in (94)] poses a promising approach for optimizing CAR T-cell therapy in the foreseeable future. Strategies to do so, include additional genetic manipulation that, e.g., has led to the design of ROS-resistant CAR T-cells, the expansion of CAR T-cells in presence of cytokines that promote metabolic fitness such as IL-21 (95), and their combined use with agents such as adenosine receptor antagonist that target tumor metabolism-triggered detrimental effects (96).

In summary, the multifaceted metabolic alterations in B-NHL and CLL have been the subject of intense research. However, more research is needed to better understand the complex immunometabolic interactions in order to help us to further improve the efficacy of emerging immunotherapies such as CAR T-cells or immune cell engaging antibodies.

## Author Contributions

All authors listed have made a substantial, direct, and intellectual contribution to the work and approved it for publication.

## Funding

RB, AM, and DM were funded by the Deutsche Forschungsgemeinschaft (DFG, German Research Foundation) - Project-ID 324392634 - TRR 221.

## Conflict of Interest

The authors declare that the research was conducted in the absence of any commercial or financial relationships that could be construed as a potential conflict of interest.
